# Role of noise and parametric variation in the dynamics of gene regulatory circuits

**DOI:** 10.1038/s41540-018-0076-x

**Published:** 2018-11-05

**Authors:** Vivek Kohar, Mingyang Lu

**Affiliations:** The Jackson Laboratory, 600 Main St, Bar Harbor, ME 04609 USA

## Abstract

Stochasticity in gene expression impacts the dynamics and functions of gene regulatory circuits. Intrinsic noises, including those that are caused by low copy number of molecules and transcriptional bursting, are usually studied by stochastic simulations. However, the role of extrinsic factors, such as cell-to-cell variability and heterogeneity in the microenvironment, is still elusive. To evaluate the effects of both the intrinsic and extrinsic noises, we develop a method, named sRACIPE, by integrating stochastic analysis with random circuit perturbation (RACIPE) method. RACIPE uniquely generates and analyzes an ensemble of models with random kinetic parameters. Previously, we have shown that the gene expression from random models form robust and functionally related clusters. In sRACIPE we further develop two stochastic simulation schemes, aiming to reduce the computational cost without sacrificing the convergence of statistics. One scheme uses constant noise to capture the basins of attraction, and the other one uses simulated annealing to detect the stability of states. By testing the methods on several synthetic gene regulatory circuits and an epithelial–mesenchymal transition network in squamous cell carcinoma, we demonstrate that sRACIPE can interpret the experimental observations from single-cell gene expression data. We observe that parametric variation (the spread of parameters around a median value) increases the spread of the gene expression clusters, whereas high noise merges the states. Our approach quantifies the robustness of a gene circuit in the presence of noise and sheds light on a new mechanism of noise-induced hybrid states. We have implemented sRACIPE as an R package.

## Modeling intrinsic/extrinsic noises in gene regulatory circuits

Gene regulatory circuits (GRCs) regulate many biological processes including cell cycle, cell differentiation, and phenotypic switching. Stochasticity in the gene expression impacts the dynamics and functions of such GRCs. Vivek Kohar and Mingyang Lu from Jackson Laboratory have developed a systems-biology modeling method *s*tochastic *ra*ndom *ci*rcuit *pe*rturbation (sRACIPE), which takes the GRC topology as the only input, and simulates an ensemble of models with random kinetic parameters at multiple noise levels. Statistical analysis of the generated gene expressions reveals the basin of attraction and stability of various phenotypic states and their changes associated with intrinsic and extrinsic noises. Application of the method to single cell expression data from synthetic circuits and epithelial-mesenchymal transition in squamous cell carcinoma shows its potential in yielding new insights on the structure and function of gene regulatory networks.

## Introduction

Noise or stochastic fluctuations in molecular components have been shown to play important role in many biological processes,^1–3^ such as phenotypic switching and gene expression coordination in cell differentiation and cell cycle,^4^ in both prokaryotic^5^ and eukaryotic organisms.^3,4,6,7^ Noise can propagate in a gene network with a cascade of circuit motifs, and the expression level of a gene can vary up to six orders of magnitude between cells.^1,8^ On the one hand, processes in gene regulation induce noise in the expression of transcripts or proteins, owing to factors such as transcription bursting and low copy numbers;^1,8,9^ on the other hand, stochastic gene expression can influence the dynamics of biological systems^8^ or even create new dynamical features like oscillations, bistability etc.^1,10–13^ It is not hard to imagine that, through evolution, cells eventually learn to use gene expression noise for their own advantage. For example, noise-induced cell-to-cell variability in protein levels in an isogenic cell population allows cells to assume different, functionally important and heritable fates.^3,14^ This heterogeneity in clonal populations of cells may be essential for many biological processes as it enables the cells to respond differently to inducing stimulus.^1^ Conversely, heterogeneous individuals in different environments can produce the same cell phenotype through phenotypic buffering/capacitance.^8^

Previous studies have unveiled many features of stochasticity in gene expression,^15,16^ yet a comprehensive and quantitative understanding of the noise-induced dynamics is still elusive.^17^ Various mathematical frameworks^18–20^ have been developed to model the dynamics of gene regulatory circuits (GRCs) governing cellular processes. Here, a GRC is a functional regulatory network motif, composed of a small set of interconnected regulators. To study the stochastic dynamics of gene circuits, various simulation schemes have been developed, including stochastic simulation algorithms (SSA, such as Gillespie algorithm^21^), methods solving stochastic differential equations (SDEs), asynchronous random Boolean network model,^22^ hybrid methods that capture the multiscale nature of different types of noise^24^ and incorporate stochasticity in both discrete and continuous variables.^23^ However, most of these methods require a fixed set of kinetic parameters that are associated with the regulation of individual genes, such as production rates, degradation rates, and binding/unbinding rates of protein–DNA (dis)association. Unfortunately, it is very hard to measure these parameters directly from experiments,^18^ and therefore it limits the accuracy and prediction power of the traditional simulation schemes.

We have recently developed a systems-biology modeling method, named random circuit perturbation (RACIPE),^25^ to deal with this long-lasting issue of parameter uncertainty. RACIPE takes the GRC topology as the only input, and generates an ensemble of models with random kinetic parameters. Then conventional ordinary differential equation based simulation is used for each random model to obtain steady-state gene expression. Finally, statistical analysis is performed on the in silico gene expression data from all the models to obtain the robust features. From our previous tests on simple GRC motifs and the biological regulatory circuits governing epithelial-to-mesenchymal transition (EMT)^25^ and B-cell development,^26^ etc., we found that the steady-state solutions from an ensemble of random models form several distinct clusters according to their expression patterns, which correspond to the functional states of the circuits (e.g., the functional states A^ON^B^OFF^ and A^OFF^B^ON^ for a toggle switch with two genes A and B). The spread of the parameters of the models in a particular cluster can be associated with the robustness of the functional state against the parametric perturbation.

As the original RACIPE is based on deterministic analysis, it cannot characterize the stochasticity of gene expression. To facilitate the stochastic analysis, here we present a modeling method that integrates stochastic methods with RACIPE. Compared to existing methods, this method has the following advantages. First, the stochastic RACIPE (sRACIPE) provides a holistic picture to evaluate the effects of both the stochasticity in cellular processes and the parametric variations. Typically the noise in cellular processes is regarded as “intrinsic” if it is caused by the stochastic nature of transcriptional, translational, and post-translational regulations due to either low copy number of molecules or slow switching among the states of promoter structure, chromatin epigenetics, or nuclear architecture.^8^ If the noise is due to pathway-specific or global differences in the abundance of cellular components, or due to differences in the timing of cell-cycle events, it could be considered as “extrinsic”.^27,28^ Segregating the effects of “intrinsic” and “extrinsic” noises in gene expression is not straightforward and is being actively studied.^1,29^ Our randomization-based method, sRACIPE, captures the effects of both the intrinsic and extrinsic noises as it incorporates both the stochastic fluctuations and the parametric variations. Second, sRACIPE allows us to evaluate the effects of noise on the cellular states of a GRC. In conventional mathematical modeling, a cellular state is defined as a stable steady state (fixed point) of a nonlinear dynamical model. However, when the signaling of the system alters, the corresponding fixed point shifts accordingly. Therefore, it is particularly difficult to associate different steady states to a cellular phenotype. To deal with this issue, we define a distinct cellular state as one of the clusters of steady-state gene expression profiles from random models.^26^ With sRACIPE, we can evaluate how gene expression noise affects the formation of the clusters and the changes in their expression patterns. Third, the stochastic analysis can quantify the relative stability of the steady states for systems allowing multiple states. This is especially hard in the original RACIPE, where the deterministic analysis is adopted to solve the rate equations and where every stable steady state was considered equally probable.

To integrate the stochastic analysis with RACIPE, we have to address an important challenge as described below. Typically, one starts from an initial condition and runs stochastic simulation for a long time to obtain the steady-state probability distribution and transition rates. In RACIPE, we generate a large (~ 10^4^–10^6^) number of random models and this stochastic simulation scheme will have a very high computational cost. Moreover, each model has a distinct set of kinetic parameters; therefore, the convergence of one model does not necessarily imply the convergence of another. A good simulation scheme has to be designed to reduce the computational cost without sacrificing the convergence of statistics.

**Fig. 1:**
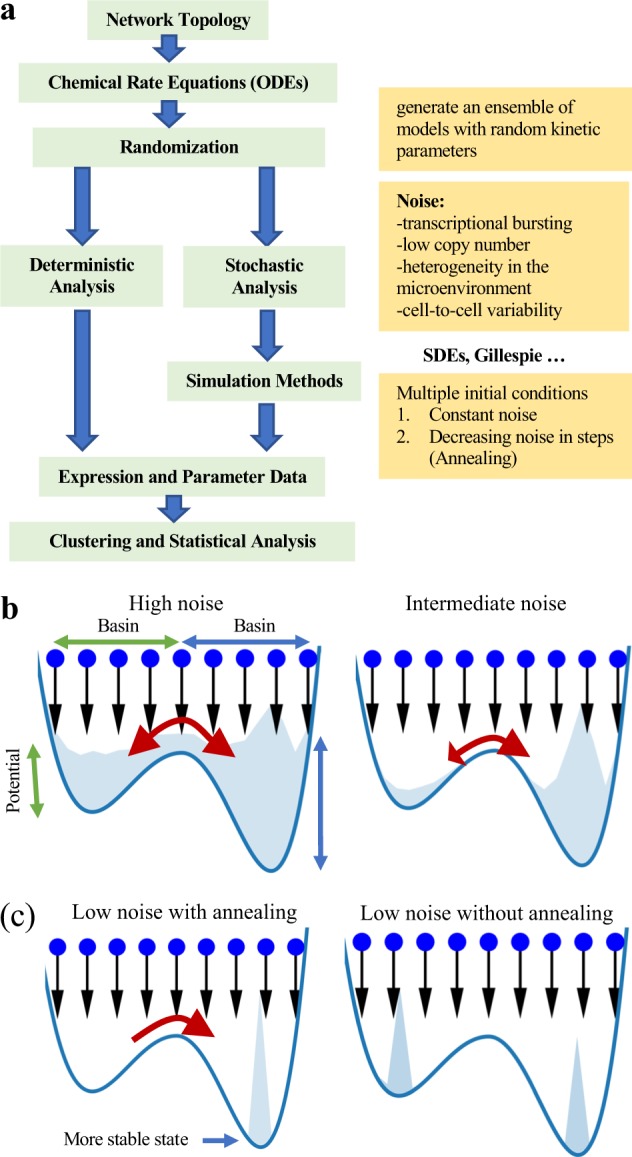
Illustration of sRACIPE. **a** The workflow of sRACIPE. The method integrates two ensemble-based sampling schemes—constant noise with multiple initial conditions (MIC) and simulated annealing (SA). **b** Illustration of the stability and basin of attraction using an example of a double-well potential. Upper left: High noise enables frequent transitions between the minima, and the steady-state probability distribution in each well is proportional to the stability of the well. Upper right: Intermediate noise permits a larger number of transitions from the less stable well to the more stable well, and some trajectories are trapped in the more stable well. Bottom left: As the noise is decreased further, the annealing based sampling scheme results in more occurrences of the particles in the more stable well. Bottom right: For low noise cases, the transitions between wells are rare. Thus, each well traps all the particles in its basin of attraction, and the steady-state probability distribution is proportional to the basin width of the wells

In the following, we will introduce the stochastic analysis methods employed in sRACIPE. We will first describe two simulation schemes—a constant noise-based method to estimate the basin of attraction of various states and another simulated annealing (SA) based method to compare the relative stability of different states and find the most stable state of GRCs. We will illustrate the methods using the canonical double-well potential system. Afterward, we will apply sRACIPE on a toggle switch, circuits with coupled toggle switches and an EMT network.^25,30^ We will demonstrate how the parametric variation and noise influence the functions of GRCs by both model simulations and analysis of single-cell gene expression data. The workflow of the sRACIPE method is presented in Fig. 1a.

## Results

### Sampling schemes for stochastic analysis

The temporal dynamics of a gene circuit can be obtained through numerical simulations of SDEs or Gillespie/kinetic Monte Carlo algorithms. A standard approach is to start with a random initial condition, run the simulation at a constant noise level for a long time, and record the state variables at equidistant time points. The histogram of these state variables gives the steady-state probability distribution of the system. Here, we refer to this sampling method as single initial condition (SIC) sampling scheme.

For a system with multiple minima and a low noise level, the SIC method converges slowly as the system gets trapped in a local minimum.^31^ To address it, we can instead perform statistics on an ensemble of simulations. Here, the method performs multiple simulations for a short simulation time starting from different initial conditions, and then it records the state variables only once at the end of each simulation. This approach, referred to as multiple initial conditions (MIC) sampling scheme, has three advantages: (1) it can simultaneously sample multiple configurations of the system, therefore providing better coverage; (2) it can be naturally integrated into RACIPE as RACIPE is also an ensemble-based method; (3) it can be easily parallelized as each initial condition evolves independently of others. Indeed, MIC and its variants^32,33^ have been adopted in simulations of equilibrium systems, but it is nontrivial for non-equilibrium systems.^32^ However, in the low noise scenario, while MIC can sample multiple configurations (thus basins of attraction^34,35^), each of the trajectories is still trapped in a local minimum; therefore, it does not estimate the stability of the minima.

Here, we propose another sampling scheme based on SA^36^ to investigate the stability of a system. This sampling scheme also generates an ensemble of simulations using multiple initial conditions. Each simulation starts with a random initial condition and a large noise level. Then, a constant noise simulation is performed for relaxation, and the state variables are recorded. The corresponding histogram of state variables from the ensemble of simulations gives the steady-state probability distribution at that noise level. After the initial stage, the noise is reduced to a slightly lower level. Here, the states obtained from the simulations of the previous noise level provide a good estimate of the initial conditions for the simulations at the next noise level. This procedure is repeated till the system reaches zero noise. The simulations from the whole protocol produce steady-state probability distributions at various noise levels. The initial high noise allows the simulations to adequately sample multiple minima, while the intermediate to low noise levels allow more transitions from less stable minima to more stable minima, eventually reaching the most stable state (Fig. 1b). In the Methods section, we describe how we integrated the sampling schemes into RACIPE. In the following sections, we will show how we tested the sampling schemes to study the stochastic dynamics of GRCs.

### Comparison of the sampling schemes in double-well potentials

We first tested the three schemes in the canonical double-well potentials (analytical functions in SI). Calculation of such potentials for GRCs is usually difficult and computationally intensive.^37,38^ Tests were performed on four variants of double-well potentials, where each variant differs from others in terms of the basin width and/or stability of wells. In SIC, the histogram of the particle positions was obtained from the positions at equidistant time points from a long simulation at a specific noise level. In MIC, the histogram was generated from the final positions of multiple short simulations for a fixed noise. In the SA scheme, histograms for different noise levels were obtained from the final positions of all the short simulations for the corresponding constant noises during SA.

**Fig. 2:**
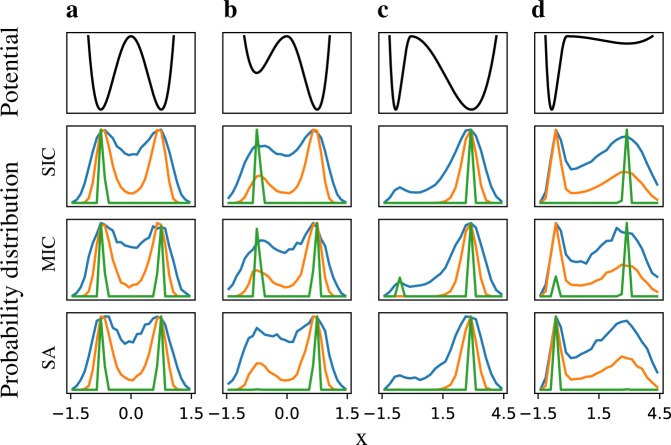
Tests of the stochastic analysis methods using double-well potentials. First row shows four variants of double-well potentials. **a** The two wells have identical stability as well as the basins of attraction; **b** the left well has lower stability than the right well, but their basins are same; **c** two wells have same stability but asymmetric basins with the right well having a larger basin, and **d** the two wells differ in stability as well as basins such that the left well is more stable but has a smaller basin of attraction. The 2nd–4th rows show the histogram of the steady states for different sampling schemes, namely, single initial condition (SIC, 2nd row), multiple initial conditions (MIC, 3rd row) and simulated annealing (SA, 4th row) for each of the four potentials and at three different noise levels—high (blue), intermediate (orange) and low noise (green). All of the methods converge for the high and intermediate noise levels whereas, for the low noise, the particle is trapped in a random well in the SIC scheme, MIC captures the basin of attraction of the wells and SA captures the most stable well. The equations, parameter values and figures for other noise levels (Fig. S1) are available in SI

In Fig. 2, for each potential variant (1st row), the 2nd–4th rows show the corresponding steady-state probability distributions at different noise levels using SIC, MIC, and SA, respectively. At high (blue curves) and intermediate (orange curves) noise levels, the probability distributions from all the methods converge in all the four variants, as noise is large enough to induce sufficient transitions between the two states. However, at low noise levels (green curves), a single trajectory is trapped in one of the basins. Thus, SIC, unlike the ensemble-based methods MIC and SA, never yields a converged distribution for all the variants. For the symmetric double-well potential (Fig. 2a, Fig. S1), both MIC and SA yield same probability distributions in all the cases. When the two wells have same basins of attraction but different stability (potential), MIC provides equal probability in both wells but SA identifies the more stable well (Fig. 2b, Fig. S1). If the two wells differ in their basins of attraction but have same minimum potential values (Fig. 2c, Fig. S1), the probability distributions obtained from MIC are proportional to their basins of attraction. However, SA has all the probability in the well with the larger basin. Lastly, when one well has larger basin width and the other is more stable (Fig. 2d, Fig. S1), SA correctly yields all the probability in the more stable well (supplementary video), whereas the probability distribution from MIC is proportional to the basin width. Altogether, our tests demonstrate that MIC and SA complement each other, especially for low noise cases, when MIC better estimates the basin of attraction and SA better estimates the stability.

### Expression noise induces state merging

**Fig. 3:**
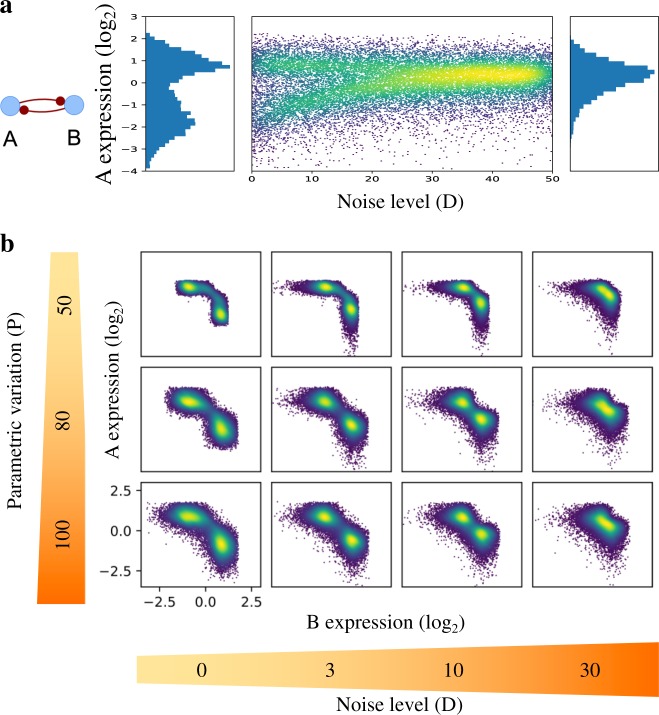
Application of sRACIPE to a toggle switch circuit. The stochastic analysis was performed using the MIC scheme. **a** The circuit diagram is illustrated on the left. The middle panel shows the heatmap of steady-state expression levels of gene A at different noise levels. The histogram on left shows the two distinct states for low noise (D<1), and the histogram on the right shows a single state for high noise (49<D<50). **b** Heatmaps of the normalized gene expression levels of the two genes for different parametric variations and noise levels. The parametric variation increases from top to bottom and the noise level increases from left to right. Two distinct clusters observed at low noise merge into a single new cluster at high noise. Parametric variation increases the spread of the clusters but does not affect their relative positions

In the above sections, we have described two ensemble-based sampling schemes for stochastic analysis. In the Methods section, we further introduce sRACIPE, which integrates MIC and SA sampling schemes with RACIPE. We applied sRACIPE to a toggle switch GRC consisting of two mutually inhibiting genes (Fig. 3a, the rate equations shown in SI). Here, MIC was used to obtain the gene expression profiles for an ensemble of models. To obtain features at different noise levels, we considered the noise level as an additional model parameter and randomized it from a uniform distribution ranging from 0 to 50. Figure 3a shows the 2D histogram of the normalized gene expression at different noise levels. At low noise levels, we observe two distinct clusters or states, as evident from the histogram on the left showing the distribution of the expressions of gene A for noise levels between 0 and 1. The distribution is similar to that from the deterministic analysis. As the noise levels increase, the two states merge, and we find a single peak in the distribution of gene expressions for noise levels between 49 and 50 (the histogram on the right in Fig. 3a). This observation of state merging can be explained as follows. When the noise increases, the contribution of noise on gene expression exceeds the contribution of the regulatory interactions. Therefore, the circuit under high noise does not have the two distinct states anymore; instead, the only state left has similar expression of both genes. Since the two clusters are symmetric, both MIC and SA produce the same results.

Here, we treated the noise level as a control parameter and evaluated the changes in gene expression. In a sense, this analysis can be considered as a global bifurcation analysis. Unlike traditional bifurcation diagram, where one alters a single parameter and keeps the other parameters constant, this global bifurcation analysis considers variations from the other parameters as well. Thus, sRACIPE method has the potential to provide global pictures of systems under the control of one parameter, which in this case is the noise level. Similar analysis can be performed for any other parameter (Fig. S3).

### Differential roles of noise level and parameteric variation

We further explored the behavior of the toggle switch GRC by changing both the noise level and parametric variation (see Methods for the definition). Using both the noise level and parametric variation as two control parameters, we can plot a global 2D bifurcation diagram, as shown in Fig. 3b. We observe that, while an increase in the spread of parameters around a median value increases the spread around the two states, an increase in noise level brings the states closer, and eventually, for large noise levels, the two states merge. This new state is different from the two states obtained from the deterministic analysis (when noise is zero) and corresponds to the previously unstable state in which both genes are expressed. These results are consistent with previous studies in that gene expression noise can create new states of a GRC.^5,10,12,15,28,39–42^ We demonstrated this point by sampling a large space of parameters and systematically evaluating the circuit behaviors. Moreover, our results indicate differential roles of the parametric variation and expression noise in influencing circuits’ behavior.

### Application of sRACIPE to complex GRCs

**Fig. 4:**
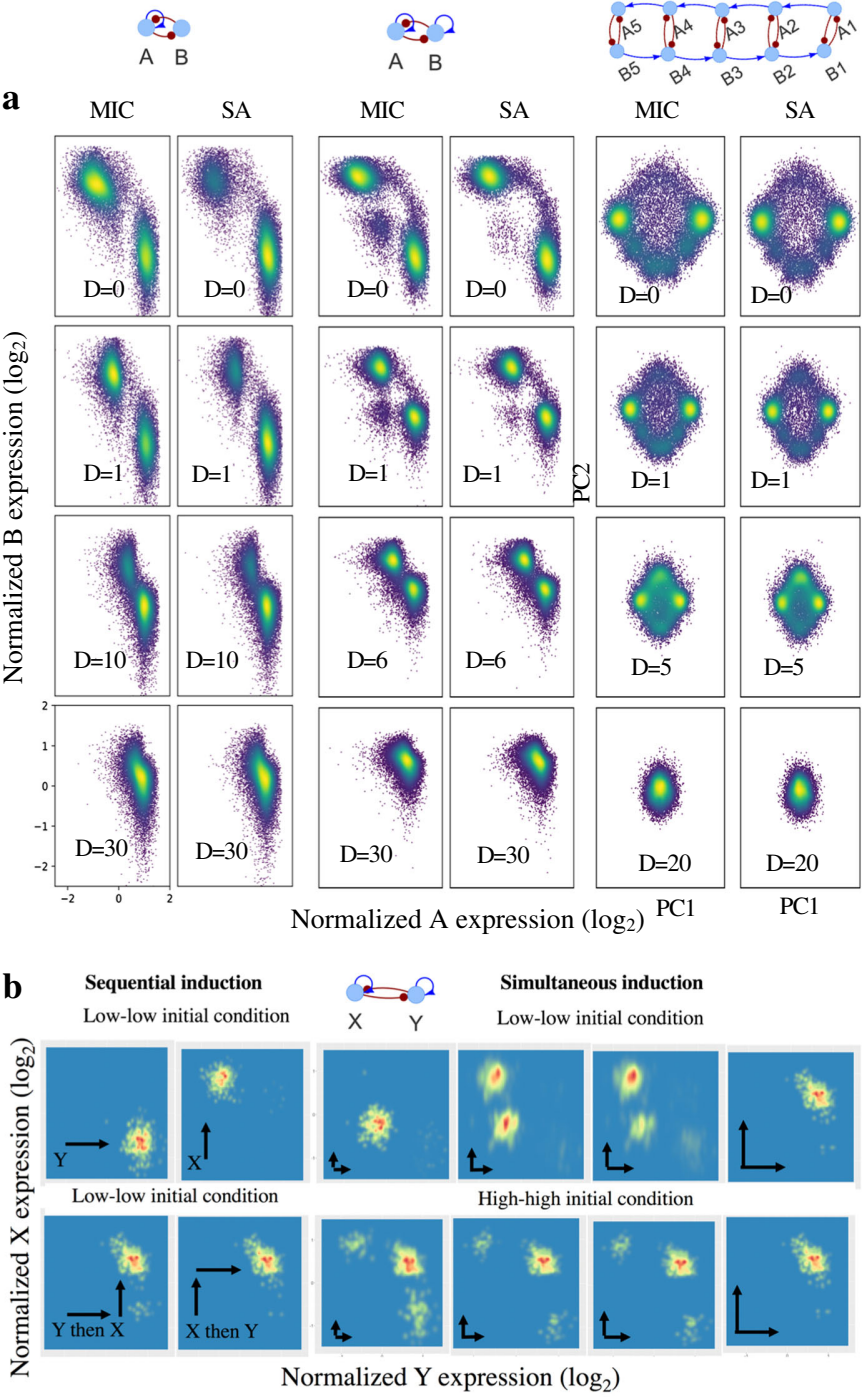
Application of sRACIPE to toggle-switch-like circuits. **a** Normalized gene expressions for several toggle-switch-like circuits using the MIC and SA methods. When noise is low, the MIC scheme provides an estimate of the basins of attraction of the states, whereas SA identifies the most stable state. At high noise, the two methods yield similar results. The results are presented for a toggle switch in which one gene is self-activating (the leftmost panel), a toggle switch in which both genes are self-activating (the middle panel), and a circuit with five coupled toggle switches (the rightmost panel). The activating links are shown in blue and the inhibitory links are shown in red. Principal components analysis of the gene expression patterns was used for dimensionality reduction and the first two components are shown here. In all the cases, increase in noise level brings the clusters together and eventually merges them into a final state. Some clusters merge first, suggesting that they are less stable than the others. **b** sRACIPE simulations to recapitulate experimental observations of the dynamics of a toggle switch with both self-activating genes. sRACIPE was applied to an ensemble of 350 quadrastable models (see SI for details). Starting from the low–low steady state as the initial condition, the models can be driven to other steady states by enhancing the self-activation links, as shown in the heatmap of the gene expressions. The induction is applied to Y only (1st column top), Y then X (1st column bottom), X only (2nd column top), and X then Y (2nd column bottom), respectively. 3rd–6th columns show the dependence of the final gene expressions on the initial conditions—low–low (top) and high–high (bottom), when the induction of X and Y self-activations are applied simultaneously. With all models in the low–low state initially, the fraction of the low–low state decreases as self-activation inductions increase, and eventually for a large induction, most of the models reach the high–high state (top 3rd–6th columns, left to right). When the inductions are removed (bottom 3rd–6th columns, right to left), the steady-state distributions don’t change significantly, and models do not switch back to the low–low state. Induction factor (IF, see SI for details), the multiplier by which self-activation link parameters of gene X are increased to model the inductions, are chosen as 15, 15, 1,1.55, 1.8, and 15, respectively (1st–6th columns)

Next, we studied some complex circuits, i.e., a toggle switch with one self-activation link, a toggle switch in which both genes are self-activating, and a circuit with five coupled toggle switches (Fig. 4). Similar to the earlier toggle switch example, the number of states as well as the gene expression pattern of these states changes with the increase in noise levels. These circuits have more than two states (i.e., clusters) and different states merge at different noise levels, suggesting these states have different levels of stability. For example, in the toggle switch with self-activations on both genes, the third intermediate cluster merges before the merging of two larger clusters. We used both of MIC and SA to evaluate the basins of attraction and the stability of the states. Similar to the double-well potential cases discussed earlier, we observe that the number of models in the different states at high noise is similar for both MIC and SA (again indicating that both methods estimate the stability), and more stable states have a larger number of models. At low noise, the difference between the two methods can be observed prominently for the toggle switch with one self-activation, indicating different basins of attraction and stability of the two states. The difference is less evident in the symmetric cases where the two dominant states are not affected much, but the intermediate state has a lesser number of models at low noise using the SA method. In short, sRACIPE provides a global view of the dynamics of GRCs and allows the estimation of the basin of attraction by MIC and the stability by SA.

The measure of basin of attraction and stability by sRACIPE could nicely interpret recent experimental observations by Wu et al.^43^ on a synthetic toggle switch circuit with self-activations on both genes (Fig. 4a, the middle circuit). Wu et al. found that this circuit can exhibit four distinct states where the expression of the two genes are low–low, low–high, high–low and high–high. From the experiments, the synthetic circuit initially resides in the low–low state. Increasing the strength of the self-activations for both genes by drug inductions drives the circuit from the low–low to high–high state. The order of the inductions determines whether the circuit goes from low–low to high–high through the low–high or high–low state (Fig. 4b 1st and 2nd columns).

To better recapitulate the circuit’s dynamical behavior, we applied sRACIPE to generate an ensemble of random models, from which we selected all of the quadrastable models for further analysis. Using both MIC and SA at zero noise limit (Fig. S2), we found that the basin of attraction of the low–low state is much larger than that of the high–high state; whereas the high–high state is much more stable, as no model was found in the low–low state after annealing. We adjusted the parameter ranges of the models (see SI) and found that the simulations work well in the low noise limit (Fig. 4b). Indeed, the noise in the system cannot be large, as no multiple states were observed in the absence of drug inductions and the initial low–low state is a stable state. Interestingly, our simulation results can explain the finding^43^ that, when the inductions are removed from the high–high state, the models continue to stay in the high–high state even when the parameters are back to the values used before the induction. To the best of our knowledge, this difference in the basin and stochastic stability of the low–low and high–high states has not been studied earlier, and our sRACIPE framework has an advantage over traditional simulation methods to analyze these features.

### Quantification of GRC’s robustness

**Fig. 5:**
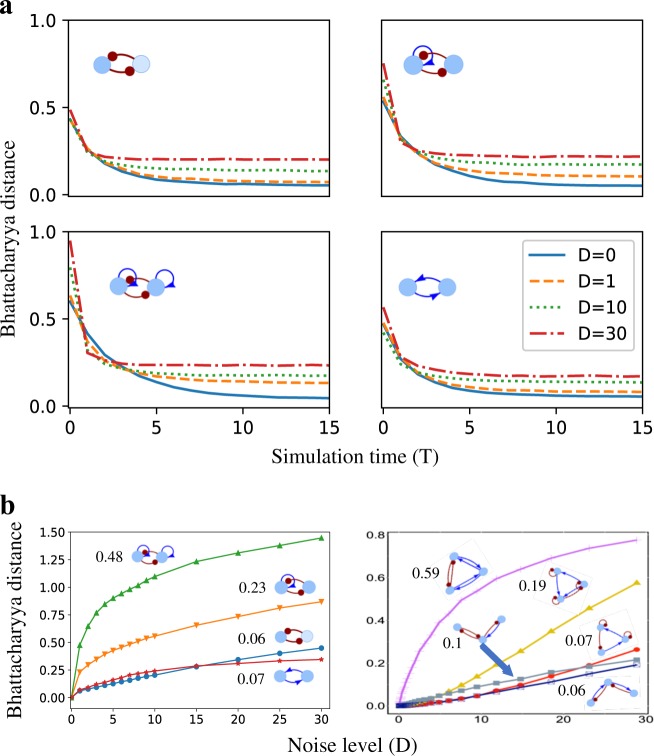
Response time and noise robustness of gene regulatory circuits **a** Tests were performed on a toggle switch circuit. The Bhattacharyya distance (BD) was calculated between the probability distribution of the gene expression sampled at the end of the simulations (simulation time, *T* = 50) for 10^6^ models at zero noise (*D* = 0) and the probability distributions at different time points during the simulations for different noise levels. The BD value for *D* = 0 approaches zero after *T* = 15, indicating that the models have converged to steady-state solutions. Simulations with larger noise converge faster, though BD is larger. **b** The response of BD with respect to the noise levels for toggle-switch-like circuits (left panel) and some three-gene circuits (right panel). The numbers indicate the noise robustness (BD at *D* = 1.0) of different circuits

We also observed that noise improves system’s response time, or so as to say, the time that the circuits take to reach the steady-state probability distribution decreases with the increase in the noise levels (Fig. 5a for the results of the toggle switch GRC). Here, we compared the probability distributions at multiple time points to the probability distributions at the end of the simulations by the Bhattacharyya distance (BD, details in SI Methods) between them. Saturation in the BD values implies that the system has relaxed and converged. At higher noise levels, there is more variability in the steady-state distributions, so the saturated BD values are larger for higher noise levels. But the system reaches this saturated BD value in shorter simulation time. Further, we found that self-activating switches have larger BD than switches without self-activations (Fig. 5b), indicating that circuits with self-activating loops are less robust against noise. Figure 5b shows the BD curves for different noise levels and the robustness of the circuit against noise (R_D_ values, see Methods for the definition) for several toggle-switch-like circuits and some three-node circuits.

### Application to a GRC governing EMT

**Fig. 6:**
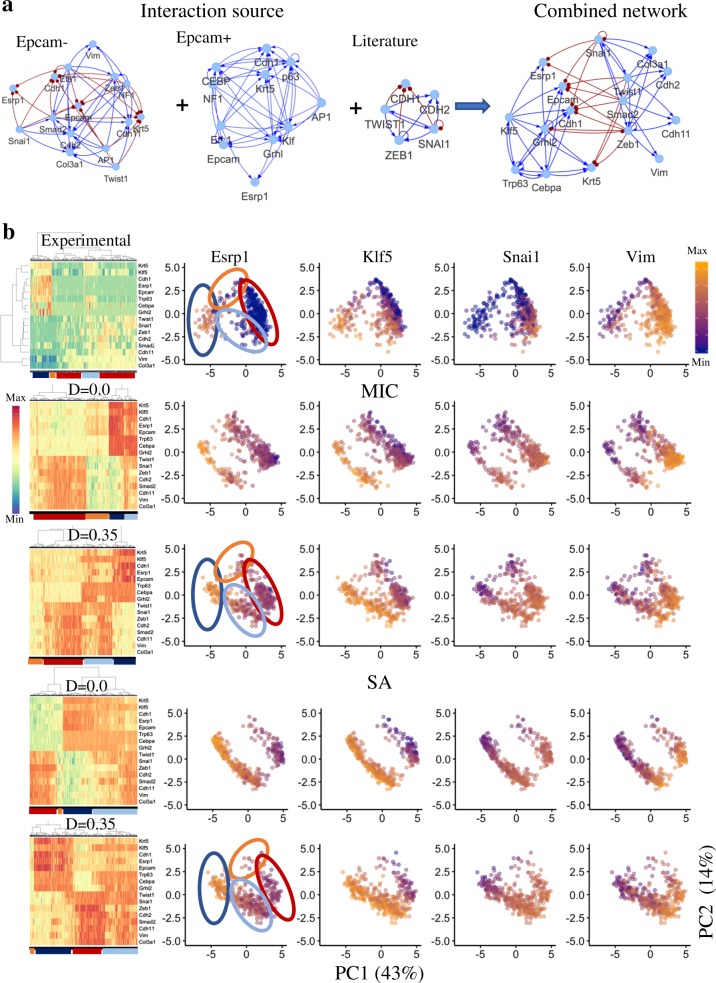
Application of sRACIPE to an EMT circuit in SCC. **a** An EMT transcriptional regulatory circuit (rightmost) was constructed by combining existing Epcam− and Epcam+ networks in SCC and the other literature data. The activating links are shown as blue lines and arrows, and the inhibitory links are shown as red lines and dots. **b** Hierarchical clustering and PCA plots of the distribution of gene expressions from the experimental data (1st row) and simulated data by MIC (2nd and 3rd rows) and SA (4th and 5th rows). The steady states corresponding to the E (dark blue) and M (red) states as well as the hybrid (light blue) and the low-expression (orange) states are marked in the Esrp1 gene expression data. The gene expression distributions in the stochastic simulations match better than the gene expression distributions from the deterministic case. PCA plots are colored by the expression levels of key marker genes for epithelial (Esrp1), hybrid epithelial (Klf5), hybrid mesenchymal (Snai1), and mesenchymal (Vim) phenotypes

Computational systems biology has been applied extensively^44–47^ to elucidate the gene regulatory mechanism of the decision making of EMT during embryonic development, wound healing and cancer metastasis.^45,46,48,49^ Here, we applied sRACIPE to an EMT gene regulatory circuit in squamous cell carcinoma (SCC) obtained by combining the recently published gene regulatory networks (Epcam+ and Epcam− networks), which integrates genome-wide transcriptional and chromatin profiling^50^ in SCC,^50^ with known interactions between EMT-related transcription factors (TFs) from previous studies.^25^ Further, we removed the TFs that have inconsistent interactions in the Epcam+ and Epcam− networks. The circuit diagram is shown in Fig. 6a.

We compared the simulation results with the single cell RNA-seq data for SCC cells undergoing EMT.^49^ The gene expressions using hierarchical clustering analysis and principal components (PC) analysis of the experimental and simulated data are shown in Fig. 6 and Fig. S6–S8 in SI. Four clusters have been marked in the PC plots of the experimental data which correspond to epithelial (E) state (dark blue ovals) with high expressions of Cdh1, Epcam, Esrp1, Krt5, Grhl2, Trp63, and Klf5, mesenchymal (M) state (red ovals) with high expression of Zeb1, Twist1, Cdh2, Snai1, Cdh11, Vim, Smad2, and Col3a1, hybrid state (light blue ovals) in which some TFs from both states are expressed, and low-expression state (orange ovals) in which all TFs have low expression. Hybrid epithelial/mesenchymal (E/M) states^30,46,49^ with mixed characteristics of collective cell migration have been found in both experiments^45,49^ as well as several computational modeling studies,^30,46^ including our previous RACIPE analysis.^25^ The cells and models with these expression states that are derived from PCA have been annotated in the hierarchical clustering plots.

Clustering of the steady states of the models in the sRACIPE simulations of the EMT network yields clusters similar to the experimental clusters (Fig. 6b). Next, we evaluate the stochastic effects on the dynamics of the EMT network. In the deterministic case, the E and M state can be easily identified but there are only a few models corresponding to the hybrid states. Moreover, there is a significant proportion of models with low expression of all genes (Fig. 6b, orange ovals). Inclusion of noise increases the proportion of models in the hybrid state and decreases the proportion of models in the low-expression state. Additionally, in the stochastic simulations, the expressions of genes Cdh1 and Epcam in the hybrid state are low which is similar to their expressions in the experimental data. The simulation results are closer to experimental observations when the SA scheme is applied instead of simulations with constant noise, as with SA there are much less models with the low-expression state. Similar to our observations for toggle switch like circuits, we find that high noise in the EMT network simulations merges the different states (Fig. S9).

We have explored possible mechanisms to stabilize the hybrid EMT phenotype in our previous studies.^46,51^ Here, we present an additional mechanism in which the hybrid EMT phenotype can be stabilized due to the increase of gene expression noise. It would be interesting to validate this hypothesis experimentally in the future. Altogether, the incorporation of stochastic effects makes the simulated gene expression closer to the experimental data and the similarity increases further using the SA scheme.

## Discussion

In this work, we have developed a computational method, named sRACIPE, to integrate stochastic analysis with the random circuit perturbation (RACIPE) method. It allows us to study the effect of both gene expression noise and parametric variation on any gene regulatory circuit (GRC) using only its topology. This method is relevant to the study of multi-stable biological processes and simulates both cell-to-cell variation and stochastic gene expression for a cell population. To facilitate sampling, we proposed two ensemble-based schemes for stochastic analysis. The two methods, MIC and SA, complement each other to provide a holistic picture, where MIC estimates the basin of attraction and SA estimates the stability. We have found that GRCs with different topology have different response times and sensitivity to noise.

Our tests show that expression noise and parametric variation have qualitatively different effects on the states of GRCs within the sRACIPE framework. Parametric variation slightly broadens the spread of the states, while high expression noise causes states to merge together. Here parametric variation refers to the spread of the parameter ranges while keeping the median constant. Note that the exact number and distributions of models in different clusters depends on the model parameters and the type of distributions from which we select the parameters, but the major features like the number of clusters remain conserved.

By sampling only one initial condition for each model, sRACIPE can easily generate as many as 10^6^ models. One major challenge is how to fully utilize such a large amount of gene expression and parameter data to analyze the robust features of a GRC. These data analysis methods can be potentially used to quantify the robustness of a GRC and evaluate how this can be associated with evolutionary fitness,^52^ estimate the Waddington’s epigenetic landscape,^53^ and predict state transitions.^38^ A better understanding of stochastic behavior can be exploited to induce desired cell states and control noise-induced transitions between different states.^54^

Both gene expression noise and parametric variation are common in biological systems.^1,4,6,7,17,27,55^ On the one hand, the Gillespie algorithm (Fig S2) has been used to model the stochastic dynamics of gene expression caused by low copy number and slow switches between gene states.^21^ On the other hand, cells of different size and microenvironment can be modeled by the same rate equations but different kinetic parameters.^56^ Our method allows the analysis of both factors, therefore being an invaluable tool to study the nature of variation in a cell population, especially with the advent of single-cell techniques.

We have found that GRCs with different circuit topology may allow similar states but differ in their sensitivity to noise, consistent with several theoretical and experimental studies.^2,57^ Biological circuits are usually robust against small noise; sometimes, they could even use noise for their functions.^1^ For example, noise can create new states or destabilize existing ones.^5,10,12,15,28,39–42^

In future, the sRACIPE framework can be extended to incorporate time-dependent variation of parameters and/or noise levels, which will shed light on the temporal dynamics of the population of cells in these conditions. It has been shown that coupling between homogenous cells in a tissue through signaling, diffusion or active transport can both increase or decrease the variability in the cells.^58^ It will be interesting to explore the effect of such coupling in the heterogenous population of cells and/or the coupling between time-dependent parameter variation and stochastic gene expression.

## Methods

### Integration of stochastic analysis into RACIPE

We introduce how sRACIPE integrates the sampling schemes with RACIPE. In the case of double-well potentials, the simulations using multiple initial conditions in MIC and SA can be considered as simulations of an ensemble of identical models using only one initial condition for each model. In contrast, the models in sRACIPE are not identical as it generates a large ensemble of random models, and each of these models is subject to a simulation scheme (either MIC or SA) using one initial condition only. We chose this scheme because of the following reasons. First, since sRACIPE generates a very large number of models, there are multiple models with similar parameters, and a collection of these models will identify most of the states. Second, as we learned from our previous studies, increasing the number of models provides better convergence of the probability distribution of the simulated gene expression data compared to increasing the number of initial conditions.^26^ Third, we have tested and found similar results when sampling multiple initial conditions for each random model (Fig S4).

In the first MIC-based sampling scheme, for each model, a short simulation is performed using a random initial condition and a fixed noise to obtain the gene expression. Such gene expressions from all the models are collected for further statistical analysis. This procedure is repeated for other noise levels. In the second SA-based simulation scheme, we first pick a random initial condition for a model and perform a simulation at a high noise level. Then, for each model, using the final gene expressions from the simulation at a higher noise as the new initial condition, we perform another simulation at a slightly lower noise level. We repeat this procedure until the noise level gradually decreases to zero (details in SI). The final gene expressions from all the models are used for further statistical analysis for the corresponding noise levels.

### Parametric variation index

The parametric variation (*P*) is defined as the spread of the parameter ranges relative to the parameter range used in the original RACIPE^25^ while keeping the median constant. *P* is measured in percentages such that the ranges are same if *P* is set to 100, and a smaller *P* implies a narrower spread of parameter values. For any given value of *P*, if the range of a parameter is set to be (*x*_min_, *x*_max_) by default in RACIPE, the new range (*y*_min_, *y*_max_) can be obtained as

1 $$\begin{array}{l}y_{\mathrm{min}} = \frac{{(x_{\mathrm{max}} + x_{\mathrm{min}})}}{2} - \frac{{\left( {x_{\mathrm{max}}- x_{\mathrm{min}}} \right)}}{2}\frac{P}{{100}},\\ y_{\mathrm{max}} = \frac{{(x_{\mathrm{max}} + x_{\mathrm{min}})}}{2} + \frac{{\left( {x_{\mathrm{max}} - x_{\mathrm{min}}} \right)}}{2}\frac{P}{{100}}.\end{array}$$

### Noise robustness index

To quantify the robustness of GRCs against noise, we define the noise robustness (*R*_D_) index of a GRC as the rate of the increase in the BD (details in SI Methods) with the increase in noise level in the low noise limit:

2 $$R_D = \mathop {{\lim }}\limits_{D \to 0} \frac{{d(BD)}}{{dD}}.$$

The larger the BD values, the lower the noise robustness.

## Code availability

sRACIPE has been implemented as an R package, freely available for academic use at https://github.com/lusystemsbio/sRACIPE.

## Electronic supplementary material

Supplementary Information

EMT network

Simulated annealing in a double well potential

## Data Availability

All the simulated data was generated using sRACIPE and can be reproduced using the vignettes in the sRACIPE code available at https://github.com/lusystemsbio/sRACIPE. The EMT network is available at the Network Data Exchange portal 10.18119/N98C7Q. The single cell data for EMT in skin SCC is publicly available from the NCBI Gene Expression Omnibus under accession number GSE110357.

## Electronic supplementary material

**Supplementary Information** accompanies the paper on the *npj Systems Biology and Applications* website (10.1038/s41540-018-0076-x).
