# Supplementary material for: Role of noise and parametric variation in the dynamics of gene regulatory circuits
Source: NPJ Syst Biol Appl. 2018 Nov 5;4:40. doi: 10.1038/s41540-018-0076-x (PMC6218471; doi:10.1038/s41540-018-0076-x)
Supplement: Supplementary file 1 — Supplementary Information [file 41540_2018_76_MOESM1_ESM.docx]

**Supplementary Information**

**Role of noise and parametric variation in the dynamics of gene regulatory circuits**

Vivek Kohar, Mingyang Lu

The Jackson Laboratory, 600 Main St, Bar Harbor, ME 04609

**SI Methods**

***1. Double Well Potential***

The equations of the double well system governing the position (x) of a particle in a bistable potential (Fig. 2 from the main article):

$\frac{dx}{dt}=2x-{4x}^{3}$ (S1)

$\frac{dx}{dt}=0.5\left( 2x-{4x}^{3} \right),$ if x<0 and $\frac{dx}{dt}=2x-{4x}^{3},$ otherwise. (S2)

$\frac{dx}{dt}=2x-{4x}^{3},$ if x<0 and $\frac{dx}{dt}=2(0.25x)-{4\left( 0.25x \right)}^{3}),$ otherwise. (S3)

$\frac{dx}{dt}=10\left( 2x-{4x}^{3} \right)$, if x<0 and $\frac{dx}{dt}=2(0.25x)-{4\left( 0.25x \right)}^{3}),$otherwise. (S4)

The corresponding potential can be obtained by integrating the equations with respect to x, for example, for equation (1), $V(x)=x^{4}-x^{2}$.

*Simulation details:*

General information regarding simulation of stochastic trajectories can be found in reference ^1,2^. In the SIC sampling scheme, we simulated the bistable systems given by Equations (S1)-(S4) using a random initial condition from a uniform distribution for a duration of T=2 ×10^5^ and recorded the position of the particle (x variable) every 2 time units (T=0, 2, 4…). In the MIC scheme, we simulated 10^4^ random initial conditions from a uniform distribution for a duration of T=1000 and recorded the x values at T=1000. In the SA method, we used 10^4^ initial conditions from a uniform distribution and 50 noise levels. The starting high noise level D is chosen to be 1 for the first three models (Eqns. (S1)-(S3)) and 2 for the last model (Eqn. S4). The noise was multiplied by a scaling factor (0.93) for subsequent levels and fixed at D=0 for the last level. Simulations were carried out for a duration of T=1000 at each noise level before recording the particle position and decreasing the noise level. The noise levels shown in Fig. 2 are D=1,0.5,0 (for Equations 1, 2, and 3 in Fig. 2A, 2B, 2C respectively) and D=1.7,1.3,0 (for Equation 4, Fig. 2d). Histograms for other noise levels are shown in Fig. S1. Note that the histograms in Fig. 2 of the main text and Fig. S1 are scaled to set the maximum value to one.

***2. RACIPE: A random perturbation approach to gene regulatory circuit modeling***

For a given circuit topology, which could be inferred from the literature, interaction databases, computational methods or a combination of these, we characterize the gene expression of the GRC under a range of chemical kinetic parameters and noise levels. For modeling a broad class of gene regulatory circuits, we map the interaction network to coupled chemical rate equations and their time evolution using deterministic simulations.

In this method, for a gene A that is regulated by two genes B and C, the dynamics is given by

$dA/dt=G_{A}Hs(B,{BA}_{0},n_{BA}, \lambda_{BA})*Hs(C,{CA}_{0},n_{CA},\lambda_{CA})-k_{A}A$ , (S5)

where A is the gene expression level of gene A, G is its production rate, i.e., the production rate of the gene when no regulator is bound to the promoter, and k_A_ is the degradation rate of gene A. H^S^ is the shifted hill function used for modeling a regulatory interaction from B to A and is given by,

$Hs\left( B,{BA}_{0},n_{BA},\lambda_{BA} \right)={\frac{1}{K}(\lambda}_{BA}+\left( 1-\lambda_{BA} \right)/(1+\left( \frac{B}{{BA}_{0}} \right)^{n_{BA}}))$ (S6)

Here BA_0_, n_BA_ and λ_BA_ are the threshold level, the Hill coefficient of regulation, and the maximum fold change of the gene B for the regulatory link going from gene B to gene A. K is a scaling factor such that K = 1 for an inhibitory link and K = λ_BA_ for an excitatory link from gene B to gene A. If there are more than one inward regulatory links on a gene, we model each of these interactions via the shifted Hill function and multiply all the Hill functions, for example, we have modeled two inward regulatory links from genes B and C on gene A in Eqn. (S5). The self-regulatory links are incorporated in the similar way, for example, for a self-regulatory link of gene A, we consider an additional link from gene A to itself.

**Table 1**

| **Parameter** | **Range (P=100)** |
| --- | --- |
| G | 1-100 |
| k | 0.1-1.0 |
| n | 1-6 |
| λ | 1-100 |

Determining the chemical kinetic parameters of the coupled equations is a hard problem with multivalued solutions and may not be feasible at all in many cases^3^. To account for this uncertainty and dynamical nature of most kinetic parameters, we sample various kinetic parameters randomly from a distribution of a given median and width. Choosing parameters from various distributions like uniform, normal, exponential or logarithmic distribution yields qualitatively similar results^4^. In this study, we used uniform distribution to select the parameters from the range given in Table 1. If the interaction between a pair of genes (for example gene A and B) is inhibitory, we take the inverse of λ_BA_ as the fold change so that fold change values are between 0.01 and 1 with a median of 0.02. We define parametric variation (P) as the spread of the parameter ranges relative to the parameter range given in the Table 1 while keeping the median constant. P is measured in percentages such that the ranges are same if P is set to 100 and a smaller P indicates a narrower spread of parameter values. For any other value of P, the new range (y_min_, y_max_) can be obtained as $y_{min}=\frac{(x_{max}+x_{min})}{2}-\frac{\left( x_{max}-x_{min} \right)}{2}\frac{P}{100}$, $y_{max}=\frac{(x_{max}+x_{min})}{2}+\frac{\left( x_{max}-x_{min} \right)}{2}\frac{P}{100}$ where (x_min_, x_max_) is the range for P=100.

The thresholds for various interactions are selected such that each gene roughly satisfies the half functional rule, i.e., the probability for a given link to stay active over all ensembles of models is close to half. To accomplish this, we first calculate the median (M^0^) of steady state values (G/k) of an isolated gene using random values for G and k, selected from the predefined ranges. In the second stage, we model only the inward links on each gene and the threshold values for these links are sampled from the interval (0.02M^0^, 1.98M^0^) where M^0^ is estimated median from the first step. From these simulations, we obtain a new steady state distribution for any gene X and calculate the median $M_{X}^{1}$ of each gene. Finally, values from (0.02$M_{X}^{1}$, 1.98$M_{X}^{1}$) are used to sample the threshold parameters for all outward regulatory links from gene X. This way, we construct a model of the core regulatory circuit by sampling each of the variables from a given distribution with fixed median and parameter variation.

Once the parameter values have been selected, we simulate the system using an initial condition selected from a logarithmic distribution whose maximum is G_A_/k_A_ and minimum is $\frac{G_{A}}{k_{A}}({}_{B}(\frac{{}_{BA}^{I}}{{}_{BA}^{E}})),$where ∏_B_ denotes the product over B, ${}_{BA}^{I}$ is the fold change of inhibitory links and ${}_{BA}^{E}$is the fold change for excitatory links. This range corresponds to the minimum and maximum possible values of expression for gene A in the deterministic analysis for the given set of parameter values. Starting with this random initial condition, we simulate the coupled ordinary differential equations for sufficiently long time to enable the system to reach a steady state and then the final gene expression values are recorded. We tested our results with both the Euler method as well as the Runge-Kutta fourth order method for numerical integration. A fifth order method with adaptive time step is also available in the accompanying R package^5^. The process is repeated a large number of times, to generate a large (ranging from ten thousand to one million) ensemble of models. The final gene expression values from this ensemble of models are used for statistical analysis where we perform principal component analysis, t-SNE embedding and hierarchical clustering analysis to identify patterns in gene expression profiles.

***3. Stochastic analysis of gene regulatory circuits***

Stochastic fluctuations are incorporated by adding a noise term to the dynamical equations such that the stochastic differential equation is

$$\frac{dA}{dt}=G_{A}Hs\left( B,{BA}_{0},n_{BA}, \lambda_{BA} \right)*Hs\left( C,{CA}_{0},n_{CA},\lambda_{CA} \right)-k_{A}A+D_{aA}\eta_{1}\left( t \right)+D_{mA}\eta_{2}(t)A$$

where D_aA_ is the additive noise level for gene A, D_mA_ is the multiplicative noise level for gene A and η_1,2_(t) are the instantaneous delta correlated noise terms with zero mean and unit variance. One can have same noise levels for all genes or a different level for each gene. We tested the numerical integration of the stochastic differential equations using both the Euler-Maruyama (EM) method as well as the weak second order Runge-Kutta method^6^ though the results presented here use the EM method. Moreover, we studied the ensemble of models using stochastic simulation analysis using Gillespie algorithm^7,8^ and obtained similar cluster states (see Fig S2).

***4. Statistical analysis of an ensemble of models***

Once we obtain all of the gene expression data, we take the logarithm (base 2) and normalize each gene independently by subtracting the mean expression level and then dividing by the standard deviation, both of which are obtained from deterministic simulations (D=0). Such a normalization step enables comparison of the gene expression at different noise levels. These normalized gene expression profiles are used for further analysis to identify clusters using principal component analysis, t-SNE, and hierarchical clustering using Euclidean distance and ward linkage.

Bhattacharyya distance (BD) was used to compare the similarity of probability distributions. BD between two distributions is given by $BD=-ln(\sum_{x} \sum_{y} \sqrt{p\left( x,y \right)q(x,y)})$, where p(x,y) and q(x,y) are the two probability distributions of variables x and y. In our case, x and y were selected as the gene expressions of two genes A and B in case of toggle switch and PC1 and PC2 from principal components analysis in the case of circuits with more than two genes. A larger BD implies a larger difference between the probability distributions and BD is zero between identical distributions.

***5. Application of sRACIPE to a synthetic circuit***

We studied a synthetic circuit, which consists of two genes with mutual inhibitions and self-activations. From the sRACIPE simulations of this circuit, we observe that this circuit can exhibit four distinct states (middle columns, Figure 4). Utilizing the experimental observations^9^, we can fine-tune the ranges of parameters for randomization. As the synthetic circuit is designed to be quadrastable, we selected the models which yielded all the four distinct states. To identify the distinct states, we simulated one million models and log transformed the normalized data and a threshold of 0.25 was selected as the boundary between the low and high state for a gene. Each model was simulated using one hundred initial conditions and if the model reached a steady-state solution in each of the four quadrants for at least one initial condition in each quadrant, then the model was classified as quadrastable. From one million random models, we found 350 quadrastable models, which were then used for further analysis. As the experimental circuit is asymmetric such that the high-low state is more prevalent than the low-high state, we multiplied the fold change of inhibitory link from gene X to gene Y by 0.05. The effect of inductions was modeled by decreasing the thresholds and fold change of self-activation links. The decrease in these parameters for gene Y self-activation was taken to be 1.5 times the decrease for gene X self-activation. We define “Induction Factor (IF)” as the ratio by which the threshold for gene X self-activation link is decreased. The modified parameters are,

$${\lambda'}_{YX}=max(1,0.05*\lambda_{YX});$$

$${YX'}_{0}=\frac{{YX}_{0}}{IF}; {XY'}_{0}=\frac{{XY}_{0}}{1.5*IF};$$

$${\lambda'}_{XX}=\max\left( 1,\frac{\lambda_{XX}}{IF} \right); {\lambda'}_{YY}=\max\left( 1,\frac{\lambda_{YY}}{1.5*IF} \right).$$

***6. Application of sRACIPE to an EMT gene Network***

*Network Construction*

We obtained the EMT circuit from Latil et al. by combining the circuits for Epcam+ Tumor Epithelial Cell (TEC) and Epcam- Tumor Mesenchymal Cells (TMC) and removing the transcription factors (TFs) AP1, Ets1, and NF1. These TFs are high in both Epcam+ TEC as well as Epcam- TMC, therefore they probably do not play the role of decision-making in the core circuit. We added additional regulatory links among the other TFs according to the literature data^4^.

*Experimental data analysis*

We retrieved the publicly available single cell RNA-seq data from NCBI Gene Expression Omnibus accession number GSE110357. The read counts from this dataset were normalized using *scran* with default parameters. For subsequent analysis, we selected the genes which are part of our network, normalized the data, and computed the PCA on this reduced dataset consisting of expression of 16 genes. Most of these genes were identified as markers of epithelial (E), mesenchymal (M) and hybrid states, and we found that these 16 genes can indeed identify the E, M and hybrid states. Fig 6 in the main text shows the PCA plots colored by the expression levels of marker genes and the hierarchical clustering (using spearman correlation as the distance and ward clustering method). As the number of cells in the experimental data is 383, we selected the data from the first 383 models from our simulations for the PCA plots. The principal components from the experimental data were used for projection of simulated data.

**SI Figures**

Fig S1 **Double well potential:** The histograms of the particle position for the four double well potentials (a) Equation S1, (b) Equation S2, (c) Equation S3, and (d) Equation S4. shown in Fig. 1 of the main text using the SIC, MIC and SA simulation schemes. The noise levels are given at the top.

Fig S2 **Gillespie algorithm results**: (a) The probability distribution heatmap for RACIPE models of toggle switch using Gillespie simulations. Randomized models with a random initial condition were simulated using the Gillespie algorithm and the gene expression values of the models after a fixed time were used for generating the probability distributions. As the number of molecules is discrete in the Gillespie method, distinct lines corresponding to low molecules numbers are visible. (b) Smoothened joint probability distribution using kernel density estimate method. (c) Normalized gene expression for the 350 quadrastable models in the mutual inhibition self-activations circuit using random initial conditions at zero noise with MIC and SA simulation schemes.

Fig S3 **Global bifurcation analysis for selected parameters**: Gene expression values of one gene in a toggle switch as the parameters are changed. The histograms on the left correspond to gene expression values for the lower ten percent values of the parameters. Similarly, the histogram on the right shows the gene expression values when the parameter is in higher ten percent range. All the other parameters are chosen randomly from the range in Table 1. The middle panel shows gene expression values when (a) production rate (G) of gene A (b) degradation rate (k) of gene A and (c) threshold of the inhibitory link from gene B to gene A is varied.


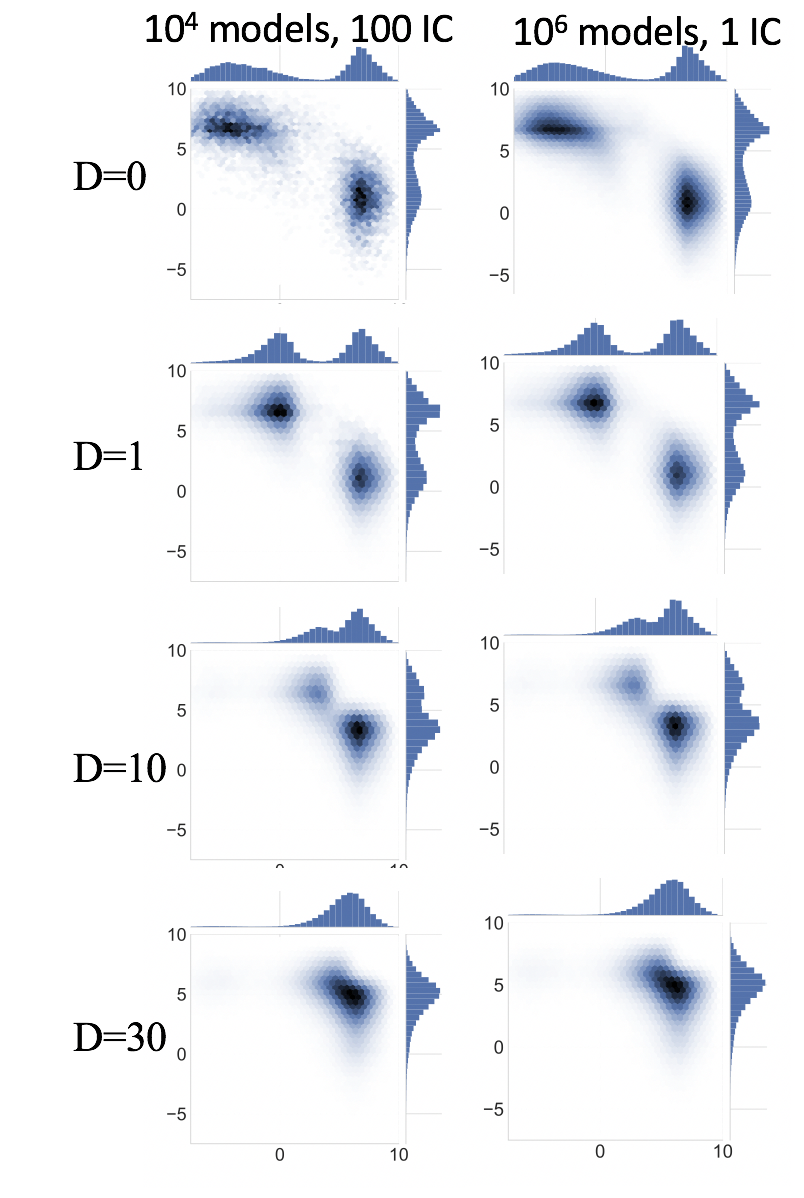


Figure S4 **Variation of the number of RACIPE models and initial conditions:** The panels show the probability distribution plots for different number of initial conditions and number of models for a toggle switch with one self-activation. The results with one initial condition and 10^6^ models are similar to (or better than, for D=0) the results for 100 initial conditions and 10^4^ models.

Fig S5 **t-SNE plots of five coupled toggle switches.** (a) Different functional states of the system form distinct clusters. (b) Combining data from noisy systems (D=5 and 20) shows that the gene expressions patterns in very noisy systems are qualitatively different from the deterministic systems.


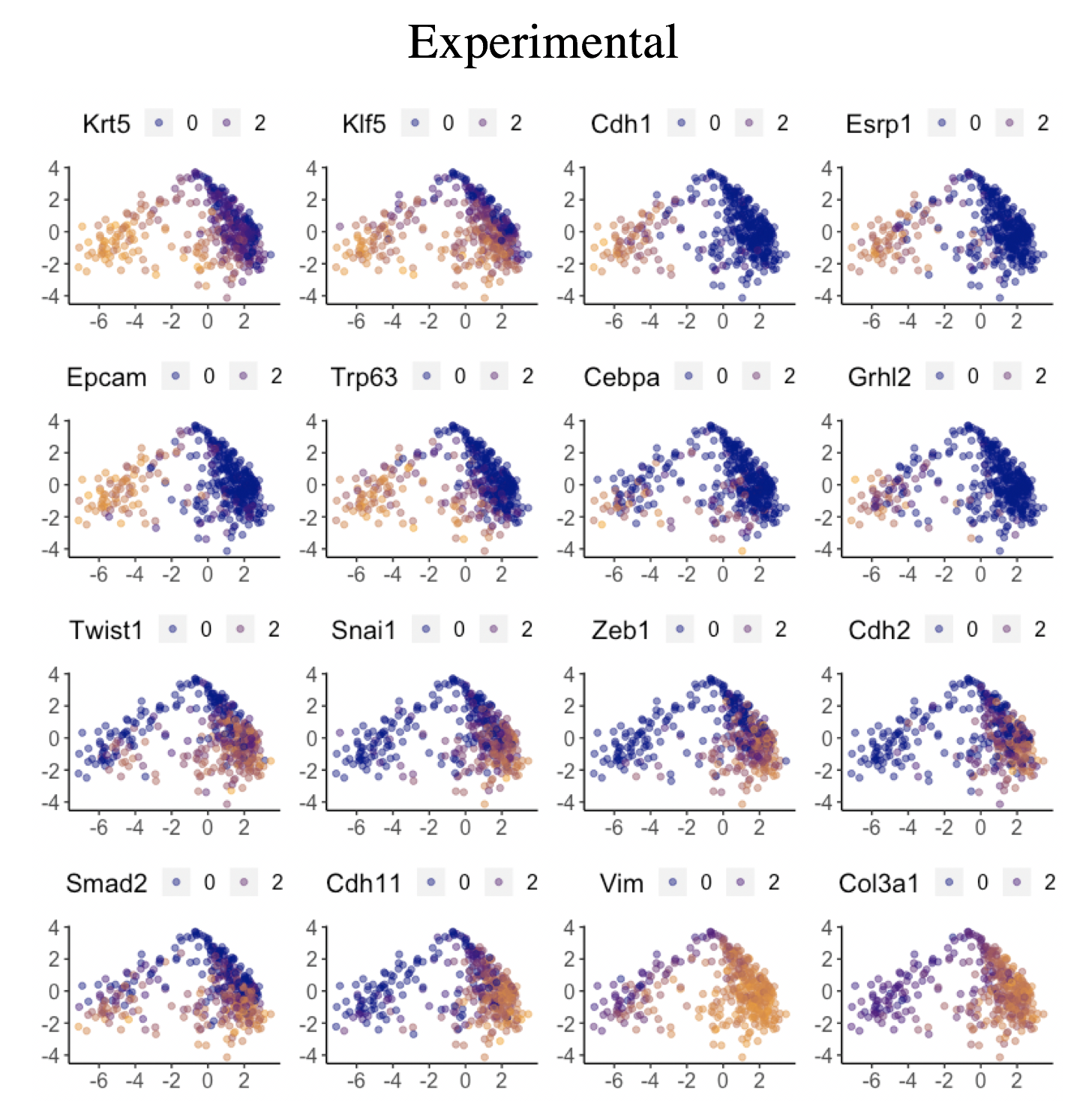


Fig S6: **Experimental single cell gene expression levels of the genes forming the EMT network**. The data were projected on the first two principal components (x-axis: PC1, y-axis: PC2) of the normalized expression levels of the 16 genes in the network. Colors in each panel represent the relative expression levels of each gene.


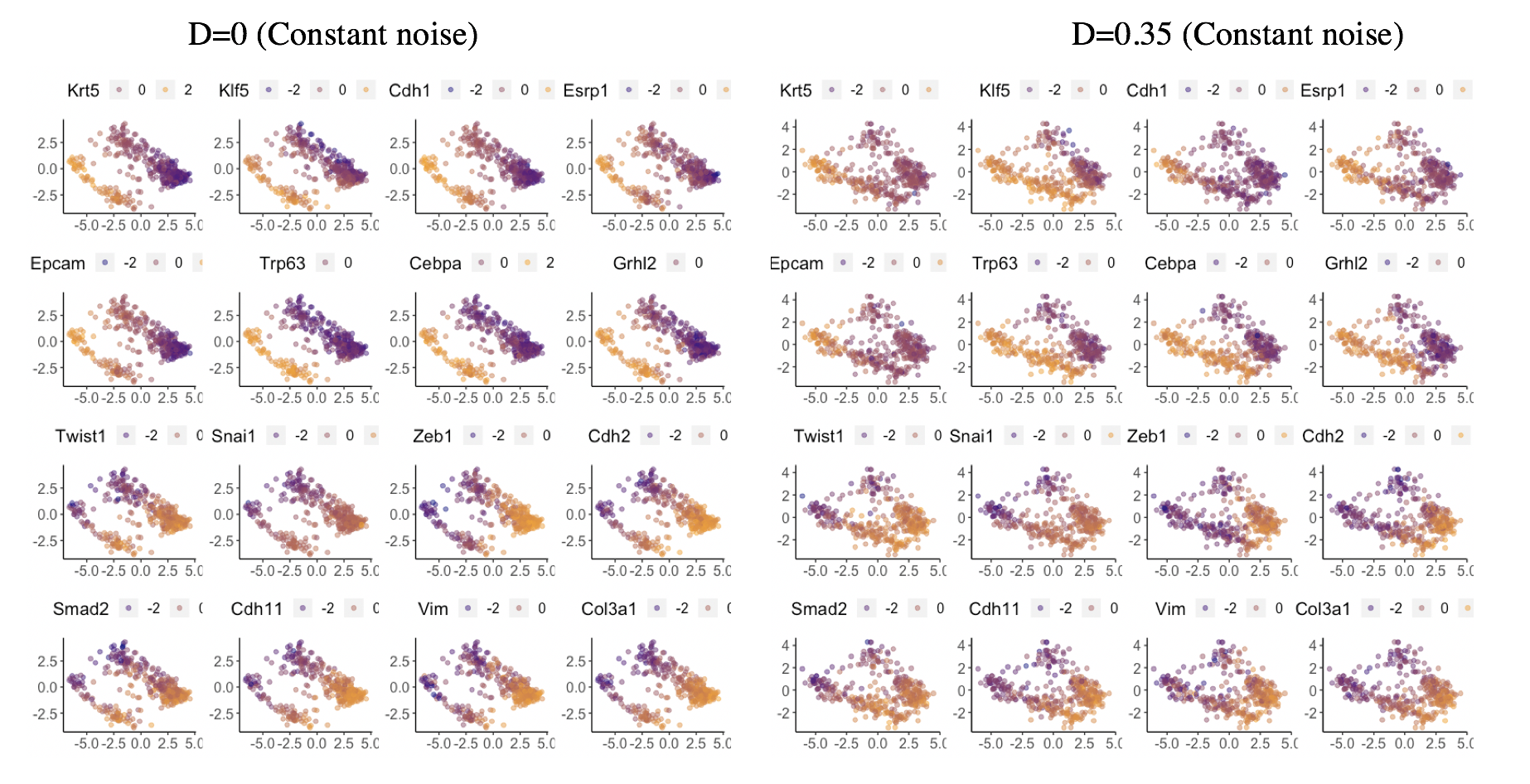


Fig S7: **Simulated expression levels of the genes in the EMT network models**. The simulated data were projected on the first two principal components of the single cell experimental data. Colors in each panel represent the relative expression levels of each gene. Here the gene expressions for both the deterministic (D=0) as well as stochastic (D=0.35) analysis using the MIC scheme are shown.


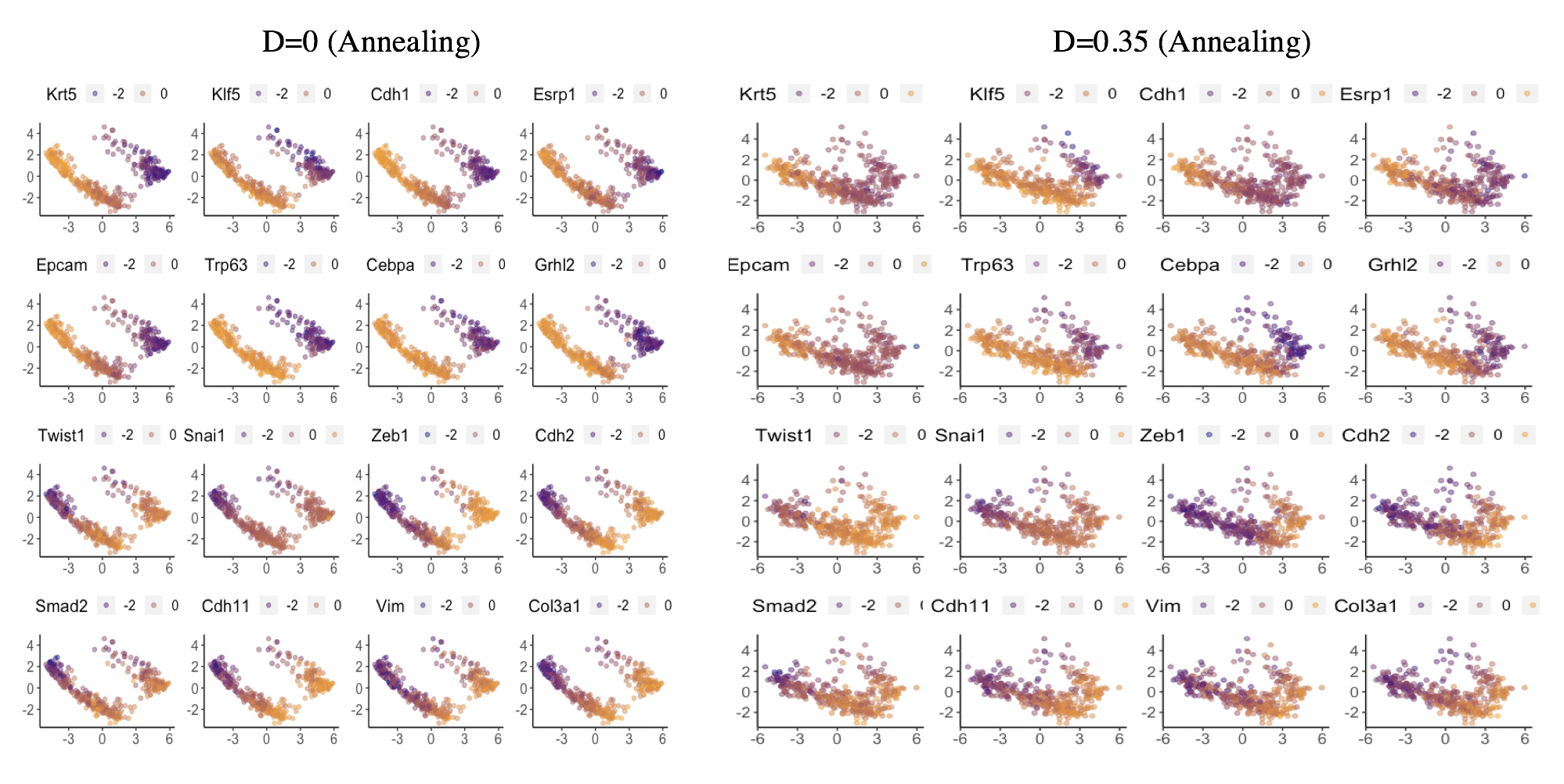


Fig S8: **Simulated expression levels of the genes in the EMT network models**. The simulated data were projected on the first two principal components of the single cell experimental data. Colors in each panel represent the relative expression levels of each gene. Here the gene expressions for both the deterministic (D=0) as well as stochastic (D=0.35) analysis using the SA scheme are shown.

Fig S9: **Simulated expression levels of the genes in the EMT network models**. The simulated data were projected on the first two principal components of the single cell experimental data at different noise levels simulated using the SA scheme. The models are colored by the relative expression level of the gene Vim.

**Reference:**

1. Hänggi, P., Talkner, P. & Borkovec, M. Reaction-rate theory: fifty years after Kramers. *Rev. Mod. Phys.* **62,** 251–341 (1990).

2. Zuckerman, D. M. *Statistical Physics of Biomolecules: An Introduction*. (CRC Press, 2010).

3. Gutenkunst, R. N. *et al.* Universally Sloppy Parameter Sensitivities in Systems Biology Models. *PLoS Comput Biol* **3,** (2007).

4. Huang, B. *et al.* Interrogating the topological robustness of gene regulatory circuits by randomization. *PLOS Computational Biology* **13,** e1005456 (2017).

5. Dormand, J. R. & Prince, P. J. A family of embedded Runge-Kutta formulae. *Journal of Computational and Applied Mathematics* **6,** 19–26 (1980).

6. Platen, E. An introduction to numerical methods for stochastic differential equations. *Acta Numerica* **8,** 197–246 (1999).

7. Gillespie, D. T. Exact stochastic simulation of coupled chemical reactions. *The Journal of Physical Chemistry* **81,** 2340–2361 (1977).

8. Gillespie, D. T., Hellander, A. & Petzold, L. R. Perspective: Stochastic algorithms for chemical kinetics. *The Journal of Chemical Physics* **138,** 170901 (2013).

9. Wu, F., Su, R.-Q., Lai, Y.-C. & Wang, X. Engineering of a synthetic quadrastable gene network to approach Waddington landscape and cell fate determination. *eLife* **6,** e23702 (2017).
