# Supplementary material for: Role of noise and parametric variation in the dynamics of gene regulatory circuits
Source: NPJ Syst Biol Appl. 2018 Nov 5;4:40. doi: 10.1038/s41540-018-0076-x (PMC6218471; doi:10.1038/s41540-018-0076-x)
Supplement: Supplementary file 2 — EMT network [file 41540_2018_76_MOESM2_ESM.docx]

| **Topology from Literature** | | |
| --- | --- | --- |
| **Source** | **Target** | **Type** |
| FOXC2 | ZEB1 | 1 |
| KLF8 | CDH1 | 2 |
| miR-101 | KLF8 | 2 |
| miR-101 | ZEB1 | 2 |
| miR-101 | ZEB2 | 2 |
| miR-101 | SNAI1 | 2 |
| miR-141 | ZEB1 | 2 |
| miR-141 | ZEB2 | 2 |
| miR-141 | TGF-beta | 2 |
| miR-200a | ZEB1 | 2 |
| miR-200a | ZEB2 | 2 |
| miR-200a | TGF-beta | 2 |
| miR-200b | ZEB1 | 2 |
| miR-200b | ZEB2 | 2 |
| miR-200b | TGF-beta | 2 |
| miR-200c | ZEB1 | 2 |
| miR-200c | ZEB2 | 2 |
| miR-200c | TGF-beta | 2 |
| miR-205 | ZEB1 | 2 |
| miR-205 | ZEB2 | 2 |
| miR-30c | SNAI1 | 2 |
| miR-30c | SNAI2 | 2 |
| miR-30c | ZEB2 | 2 |
| miR-34a | SNAI1 | 2 |
| miR-34a | SNAI2 | 2 |
| miR-34a | TWIST2 | 2 |
| miR-34a | ZEB1 | 2 |
| miR-9 | CDH1 | 2 |
| miR-9 | ZEB2 | 2 |
| SNAI1 | CDH1 | 2 |
| SNAI1 | CDH2 | 1 |
| SNAI1 | FOXC2 | 1 |
| SNAI1 | SNAI1 | 2 |
| SNAI1 | SNAI2 | 1 |
| SNAI1 | TWIST1 | 1 |
| SNAI1 | ZEB1 | 1 |
| SNAI1 | ZEB2 | 1 |
| SNAI1 | miR-34a | 2 |
| SNAI1 | miR-101 | 2 |
| SNAI2 | CDH1 | 2 |
| SNAI2 | CDH2 | 1 |
| SNAI2 | SNAI2 | 1 |
| SNAI2 | TWIST2 | 1 |
| SNAI2 | miR-101 | 2 |
| SNAI2 | miR-200b | 2 |
| TCF3 | CDH1 | 2 |
| TWIST1 | CDH1 | 2 |
| TWIST1 | CDH2 | 1 |
| TWIST1 | FOXC2 | 1 |
| TWIST1 | SNAI1 | 1 |
| TWIST1 | SNAI2 | 1 |
| TWIST1 | TCF3 | 1 |
| TWIST1 | ZEB1 | 1 |
| TWIST1 | ZEB2 | 1 |
| TWIST2 | CDH1 | 2 |
| TWIST2 | SNAI1 | 1 |
| TWIST2 | SNAI2 | 1 |
| TWIST2 | TWIST1 | 1 |
| TWIST2 | ZEB1 | 1 |
| TWIST2 | ZEB2 | 1 |
| ZEB1 | CDH1 | 2 |
| ZEB1 | CDH2 | 1 |
| ZEB1 | miR-141 | 2 |
| ZEB1 | miR-200b | 2 |
| ZEB1 | miR-200c | 2 |
| ZEB1 | miR-200a | 2 |
| ZEB1 | miR-34a | 2 |
| ZEB1 | ZEB1 | 1 |
| ZEB2 | CDH1 | 2 |
| ZEB2 | CDH2 | 1 |
| ZEB2 | miR-200b | 2 |
| ZEB2 | miR-200c | 2 |
| ZEB2 | miR-200a | 2 |
| ZEB2 | ZEB2 | 1 |
| GSC | SNAI1 | 1 |
| GSC | TWIST1 | 1 |
| GSC | FOXC2 | 1 |
| GSC | ZEB1 | 1 |
| GSC | ZEB2 | 1 |
| TGF-beta | GSC | 1 |
| TGF-beta | SNAI1 | 1 |
| TGF-beta | SNAI2 | 1 |
|  |  |  |
| **Epcam + network** | | |
| **Source** | **Target** | **Type** |
| NF1 | Klf | 1 |
| NF1 | CEBP | 1 |
| NF1 | Grhl | 1 |
| NF1 | p63 | 1 |
| NF1 | Krt5 | 1 |
| NF1 | Epcam | 1 |
| NF1 | Cdh1 | 1 |
| Ets1 | Klf | 1 |
| Ets1 | Grhl | 1 |
| Ets1 | CEBP | 1 |
| Ets1 | p63 | 1 |
| Ets1 | Esrp1 | 1 |
| Ets1 | Krt5 | 1 |
| Ets1 | Epcam | 1 |
| Ets1 | Cdh1 | 1 |
| AP1 | Grhl | 1 |
| AP1 | Klf | 1 |
| AP1 | p63 | 1 |
| AP1 | Cdh1 | 1 |
| AP1 | Krt5 | 1 |
| AP1 | Epcam | 1 |
| p63 | p63 | 1 |
| p63 | Klf | 1 |
| p63 | CEBP | 1 |
| p63 | Grhl | 1 |
| p63 | Cdh1 | 1 |
| p63 | Krt5 | 1 |
| Klf | CEBP | 1 |
| Klf | Grhl | 1 |
| Klf | p63 | 1 |
| Klf | Cdh1 | 1 |
| Klf | Epcam | 1 |
| Klf | Esrp1 | 1 |
| Klf | Krt5 | 1 |
| Grhl | Grhl | 1 |
| Grhl | CEBP | 1 |
| Grhl | p63 | 1 |
| Grhl | Cdh1 | 1 |
| Grhl | Epcam | 1 |
| Grhl | Esrp1 | 1 |
| Grhl | Krt5 | 1 |
| CEBP | p63 | 1 |
| CEBP | Cdh1 | 1 |
| CEBP | Epcam | 1 |
| CEBP | Krt5 | 1 |
|  |  |  |
|  |  |  |
| **Epcam- Network** | | |
| NF1 | Zeb1 | 1 |
| NF1 | Cdh2 | 1 |
| NF1 | Cdh11 | 1 |
| NF1 | Col3a1 | 1 |
| NF1 | Vim | 1 |
| NF1 | Krt5 | 2 |
| NF1 | Epcam | 2 |
| NF1 | Cdh1 | 2 |
| Ets1 | Zeb1 | 1 |
| Ets1 | Cdh2 | 1 |
| Ets1 | Cdh11 | 1 |
| Ets1 | Col3a1 | 1 |
| Ets1 | Vim | 1 |
| Ets1 | Esrp1 | 2 |
| Ets1 | Krt5 | 2 |
| Ets1 | Epcam | 2 |
| Ets1 | Cdh1 | 2 |
| Ets1 | Smad2 | 1 |
| AP1 | Smad2 | 1 |
| AP1 | Zeb1 | 1 |
| AP1 | Cdh2 | 1 |
| AP1 | Cdh11 | 1 |
| AP1 | Col3a1 | 1 |
| AP1 | Krt5 | 2 |
| AP1 | Epcam | 2 |
| AP1 | Cdh1 | 2 |
| Zeb1 | Cdh2 | 1 |
| Zeb1 | Cdh11 | 1 |
| Zeb1 | Krt5 | 2 |
| Zeb1 | Col3a1 | 1 |
| Zeb1 | Vim | 1 |
| Zeb1 | Cdh1 | 2 |
| Zeb1 | Epcam | 2 |
| Snai1 | Smad2 | 1 |
| Snai1 | Col3a1 | 1 |
| Snai1 | Esrp1 | 2 |
| Snai1 | Cdh1 | 2 |
| Snai1 | Cdh2 | 1 |
| Snai1 | Epcam | 2 |
| Twist1 | Epcam | 2 |
| Twist1 | Krt5 | 2 |
| Twist1 | Col3a1 | 1 |
| Twist1 | Cdh2 | 1 |
| Twist1 | Cdh11 | 1 |
| Smad2 | Esrp1 | 2 |
| Smad2 | Krt5 | 2 |
| Smad2 | Epcam | 2 |
| Smad2 | Cdh1 | 2 |
| Smad2 | Col3a1 | 1 |
| Smad2 | Vim | 1 |
| Smad2 | Cdh2 | 1 |
| Smad2 | Cdh11 | 1 |
|  |  |  |
| **EMT Network used in Simulations** | | |
| Zeb1 | Cdh2 | 1 |
| Zeb1 | Cdh11 | 1 |
| Zeb1 | Krt5 | 2 |
| Zeb1 | Col3a1 | 1 |
| Zeb1 | Vim | 1 |
| Zeb1 | Cdh1 | 2 |
| Zeb1 | Epcam | 2 |
| Snai1 | Smad2 | 1 |
| Snai1 | Col3a1 | 1 |
| Snai1 | Esrp1 | 2 |
| Snai1 | Cdh1 | 2 |
| Snai1 | Cdh2 | 1 |
| Snai1 | Epcam | 2 |
| Twist1 | Epcam | 2 |
| Twist1 | Krt5 | 2 |
| Twist1 | Col3a1 | 1 |
| Twist1 | Cdh2 | 1 |
| Twist1 | Cdh11 | 1 |
| Smad2 | Esrp1 | 2 |
| Smad2 | Krt5 | 2 |
| Smad2 | Epcam | 2 |
| Smad2 | Cdh1 | 2 |
| Smad2 | Col3a1 | 1 |
| Smad2 | Vim | 1 |
| Smad2 | Cdh2 | 1 |
| Smad2 | Cdh11 | 1 |
| Trp63 | Trp63 | 1 |
| Trp63 | Klf5 | 1 |
| Trp63 | Cebpa | 1 |
| Trp63 | Grhl2 | 1 |
| Trp63 | Cdh1 | 1 |
| Trp63 | Krt5 | 1 |
| Klf5 | Cebpa | 1 |
| Klf5 | Grhl2 | 1 |
| Klf5 | Trp63 | 1 |
| Klf5 | Cdh1 | 1 |
| Klf5 | Epcam | 1 |
| Klf5 | Esrp1 | 1 |
| Klf5 | Krt5 | 1 |
| Grhl2 | Grhl2 | 1 |
| Grhl2 | Cebpa | 1 |
| Grhl2 | Trp63 | 1 |
| Grhl2 | Cdh1 | 1 |
| Grhl2 | Epcam | 1 |
| Grhl2 | Esrp1 | 1 |
| Grhl2 | Krt5 | 1 |
| Cebpa | Trp63 | 1 |
| Cebpa | Cdh1 | 1 |
| Cebpa | Epcam | 1 |
| Cebpa | Krt5 | 1 |
| Snai1 | Snai1 | 2 |
| Snai1 | Twist1 | 1 |
| Snai1 | Zeb1 | 1 |
| Twist1 | Cdh1 | 2 |
| Twist1 | Snai1 | 1 |
| Twist1 | Zeb1 | 1 |
| Zeb1 | Zeb1 | 1 |
| Zeb1 | Grhl2 | 2 |
| Grhl2 | Zeb1 | 2 |
